# Severity of alcoholism in Indian males: Correlation with age of onset and family history of alcoholism

**DOI:** 10.4103/0019-5545.70977

**Published:** 2010

**Authors:** Pradeep R. Johnson, Saira Banu, M. V. Ashok

**Affiliations:** Department of Psychiatry, St. John’s Medical College and Hospital, Sarjapur Road, Bangalore - 560 034, Karnataka, India; Department of Community Medicine, PSG Institute of Medical Sciences and Research, Peelamedu, Coimbatore - 641 004, Tamil Nadu, India

**Keywords:** Age of onset, alcoholism, family history density, prevention, problem drinking, severity

## Abstract

**Background:**

Family History of Alcoholism and earlier Age of Onset are found to predict Severity of alcoholism. Previous Indian studies in this regard have methodological issues related to the definition of alcoholism and reliability of information obtained.

**Aims:**

To study the relationship between the Age of onset/Family History and Severity of alcoholism.

**Settings and Design:**

Consecutively admitted, 20 to 50 year old men, with alcohol-related problems at an urban teaching hospital, were recruited.

**Materials and Methods:**

After detoxification, alcohol use detection inventory test, severity of alcohol dependence questionnaire, schedule for clinical assessment in neuropsychiatry, and family interview for genetic studies were administered. Family history density was computed.

**Statistics:**

Pearson’s correlations, linear regression, and ANOVA tests were used.

**Results:**

Family history density and severity of alcoholism were positively correlated. Age of onset of initiation had a significant negative correlation with severity. The effect of family history on the rapidity of development of Problem-drinking did not reach statistical significance among those with early age of onset. The variance explained by the ‘family history status’ and ‘age of onset’ for the severity of alcoholism was similar to that reported in earlier western studies.

**Conclusion:**

This study, with enhanced methodology, using a general hospital sample of problem drinkers concludes that the age of onset of initiation is a better predictor of severity of alcoholism, than family history of alcoholism alone. Postponing the use of alcohol till the age of 25 years could be explored as a primary prevention strategy in genetically vulnerable adolescents.

## INTRODUCTION

There is a three-to-five-fold increased risk of alcoholism in the relatives of alcoholics.[1] In various international studies, individuals with a positive family history have shown to have an increased severity of alcohol dependence.[2,3] Family history method varies across studies. Some authors have attempted to study family history by using parental alcoholism alone.[4] Others have tried multigenerational models to classify alcoholism.[4,5] Turner *et al*. proposed a Family Pattern of Analysis (FPA), which explained more variance than other methods.[6] This has led to the suggestion by Zucker *et al*., that a standard measure be used to enable across studies.[7] He proposed Family History Density (FHD), which is a modification of FPA and has a weighting scheme. It is based on familial relatedness.

A lowered age of onset has also been associated with increased severity of alcoholism and later development of alcohol dependence. Evidence suggests that the early age of onset is associated with aggression, problems with law;[8] social role maladaptation, loss of behavioral control when drinking,[9] and childhood criminality.[10] Some of the reasons stated for initiation of alcohol use early in life were pressure from peer groups, experimentation, and curiosity. Varma *et al*. found that early-onset alcoholics (age at onset of alcohol dependence 25 years or less) were younger, had a larger proportion of first-degree relatives with both lifetime use and abuse/dependence of alcohol, but not of other psychoactive substances, and they had experienced a greater number of alcohol-related problems in the previous one year. They were also higher sensation seekers and tended to display aggression, violence, and general disinhibition when drinking.[11] A recent Indian study reported that the age of onset of alcohol use in a hospital based population was 18 years and the age of onset of dependence was 27 years. They also found that these subjects developed the first criteria of dependence after six years of alcohol use and then required only four years to develop the dependence syndrome according to ICD-10.[12]

The relationship between family history and age of onset with severity of alcoholism in the Indian culture is not clear. Indian studies have generally not detailed the methodology of obtaining family history[13], are carried out in deaddiction settings[13,14] and the reliability of the family history information is not provided.[15] The objective of this study is to explore whether the relationship between these variables and the severity of alcoholism can be extended to male subjects in a general hospital, where subjects with a much wider spread of severity of alcohol use may be anticipated.

## MATERIALS AND METHODS

### Subjects

We recruited male patients between the age group of 20 to 50 years, admitted consecutively for alcohol-related problems, to medical, surgical, orthopedic, and psychiatric wards in a large teaching general hospital, over a period of 14 months. This is a tertiary care hospital, catering to the rural and urban population of Bangalore and a few neighboring districts of Tamilnadu and Andhra Pradesh, as well as parts of Kerala. The study was approved by the institutional ethics review board.

### Screening

Consecutive subjects were first administered Clinical Institute Withdrawal Assessment for Alcohol (CIWA-AD) to rule out any persistent alcohol withdrawal symptoms.[16] CIWA-AD is an eight-item scale for clinical quantification of the severity of the alcohol withdrawal syndrome. Subjects scoring less than eight on CIWA-AD were then administered Alcohol Use Detection Inventory Test (AUDIT) and only subjects scoring more than a score of eight on AUDIT were included in the study.[17] AUDIT has been has been validated and widely used in India.[18] Subjects who were medically too ill to cooperate in the interview and were associated with major psychiatric illnesses, such as psychosis, dementia, amnestic syndromes, and bipolar affective disorder when screened by the Schedule for Clinical Assessment of Neuropsychiatry (SCAN), were excluded.[19]

### Assessment

**Figure 1:**
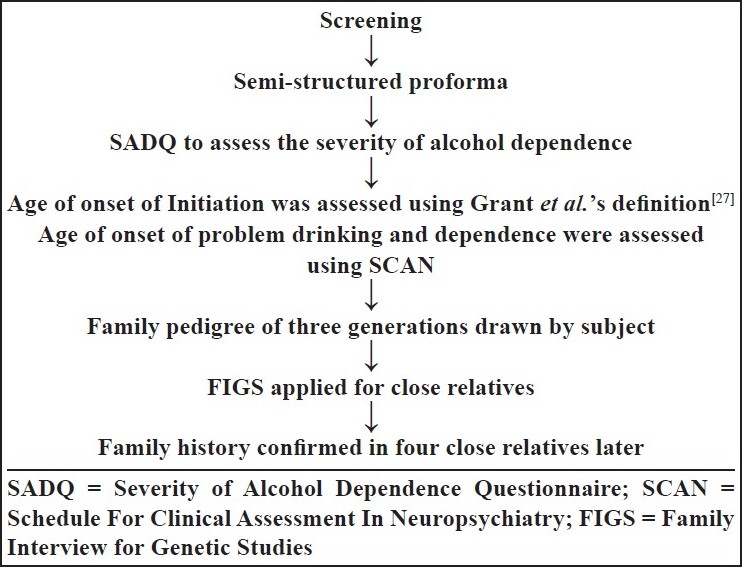
Administration of questionnaires and assessments

The subjects were then administered a semi-structured proforma and other measures in a particular sequence, in order to reduce bias [Figure 1]. We assessed the severity of alcoholism using Severity of Alcohol Dependence Questionnaire (SADQ). This is a 20-item, self-rated questionnaire, which has been developed to provide a brief and replicable method of assessing the severity of alcohol dependence. It has a high degree of test–retest reliability and a very good evidence of construct and concurrent validity.[20]

Next, we assessed the age of onset of initiation, using the definition described by Grant *et al*., which defines the age of onset of initiation, as the “age at which they first started drinking, not counting small tastes or sips of alcohol”.[21] The age of onset of Problem-drinking and dependence was assessed using the alcohol-use section of SCAN.[19] Only the age of onset of initiation and Problem-drinking was used for this study. Subsequently the age of onset was divided into two groups (early onset and late onset). Early onset consisted of subjects with the age of onset less than and equal to 25 years and late onset consisted of subjects with age of onset beyond 25 years. Similarly, age of onset of the Problem-drinking was divided into early onset (less than and equal to 25 years) and late onset (more than 25 years).

Later the subjects were asked to draw a family pedigree of three generations. The relatives were assessed for alcohol dependence syndrome using the alcohol section of the Family Interview for Genetic Studies (FIGS).[22] The data for FIGS was obtained for multiple relatives from multiple informants, but for this study we reported data on the parents and grandparents only. FIGS is an interview-guide based on the Research Diagnostic Criteria. It helps in gathering information about relatives in the pedigree to be studied. It is particularly important when the information from the subject is not reliable. It is meant to be a guide for the interviewer and enables freedom in wording the questions. Positive family history was coded when any of the six direct ancestors were found to meet the criteria for alcohol dependence syndrome and negative if none of the six direct ancestors were having features of alcohol dependence syndrome.

Family History Density (FHD) was assessed by giving weighted points to the alcoholic family members based on their relatedness to the subject (parents (0.5) and grand parents (0.25)) and non-alcoholic relatives were given a score of zero. The scores were summed over the six ancestors to obtain the FHD score, which ranged from 0–2. This information was confirmed with four close relatives of the subject, for improving the reliability of the information. The scoring system was based on familial relatedness, which was functionally the same as genetic relatedness and theoretically explicit because it took into account the influence of family environment (parents are expected to influence the probands more directly than the grandparents).[23]

### Statistical analysis

Statistical analysis was done using SPSS version 13. Descriptive statistics was used to describe the sociodemographic variables. The independent sample t test was used to assess the difference in the variables. Pearson’s correlation was used for assessing the relationship among the variables. Partial correlation was used to assess the relationship of age of onset and severity after controlling for family history density. Linear regression analysis was used for assessing the strength of association. One-way ANOVA was used to assess the significant differences in the mean severity scores after dividing the age of onset into quartiles. Among those with early age of initiation of alcohol use, Two-way ANOVA was used to analyze the influence of family history on the rapid development of Problem-drinking and severity of alcoholism.

## RESULTS

Six hundred and seventy-three patients were admitted with alcohol-related problems during the study period. Fifteen (2.22%) of these patients were female alcoholics, and 20 patients (2.97%) refused to participate in the study. Twelve patients (1.78%) were excluded from the study due to poor details from the relatives. Forty-eight patients (7.13%) had an AUDIT score of less than 8 and did not qualify for the inclusion criteria. Three hundred and seventy-eight (60.67%) patients were excluded due to acute medical complications. Two hundred subjects qualified for the study. The mean AUDIT score of the subjects was 37.37±4.61 and the mean severity score was 42.43±11.37. The mean age of onset of initiation was 21.39±5.34 years, the mean age of onset of Problem-drinking was 24.28±5.42 years, and the mean age of onset of dependence was 27.8±5.7 years. The sociodemographic variables are presented in the [Table 1]. Most of the variables such as current age, marital status, educational status, and occupational status were not significantly associated with the severity of alcoholism. Both measures of the duration of alcoholism {time to develop the drinking problem (r=−.069, *P*<.329)/dependence (r=−.091, *P*<.202) from initiation} did not correlate with the severity.

**Table 1:** Socio-demographic Profile of the Subjects

Variables	Number	Percentage
Age distribution		
20–30	33	16.5
31–40	67	33.5
> 41	100	50
Marital status		
Married	186	93
Single	14	7
Educational status		
No formal education	15	7.5
Primary	40	20
Secondary	103	51.5
Graduate	42	21
Occupational status		
Professional	23	11.5
Business	29	14.5
Farmer	22	11
Laborer	64	32
Service	22	11
Employed	35	17.5
Unemployed	3	1.5
Student	2	1
Family type		
Nuclear	175	87.5
Joint	10	5
Extended	14	7
Single	1	0.5

The difference in the mean severity scores in subjects with a positive family history (*n*=123) and negative (*n*=77) subjects was statistically significant [Table 2]. Similarly, the difference in the severity scores between subjects with early and late age of onset of initiation was also statistically significant [Table 2].

**Table 2:** Difference on the mean severity scores with the subjects divided into two groups based on each of the two variables, Family History of Alcoholism (Dichotomy) and Age of Onset of initiation (Dichotomy)

Variables	n	Severity Scores Mean (sd)	Significance (*P* value)
Family history			
Positive	123	44.59 (10.79)	0.001
Negative	77	38.96 (11.50)	
Age of onset of initiation			
Early onset (≤ 25 years)	168	43.71 (10.78)	0.001
Late onset (≥ 26years)	32	35.69 (12.18)	

Independent sample *t* test was used. n = numbers; sd = standard deviation

A positive correlation was found between FHD and severity of alcoholism (r=.259, *P*<.001). The R squared value was 0.067. This indicated that 6.7% of variance in the severity of alcoholism was explained by the FHD [Table 3]. The relationship between age of onset of initiation and severity was inverse and statistically significant (r=−.298, *P*<.001). The R square value was 0.089. This indicated that 8.9 % of the variance in severity of alcoholism was explained by the age of onset of initiation. Age of onset of initiation had a greater influence (standardized coefficient (Beta) =.249) as compared to FHD (standardized coefficient (Beta) =.197) on the severity of alcoholism when both the variables were included in the regression analysis [Table 3]. Partial correlation analysis showed that the age of onset of initiation was still associated with severity even after controlling for FHD (r=−.250, df=197, *P*<.001).

**Table 3:** Association between severity of alcoholism and predictor variables

Models	Variables	R^2^ % (variation explained by each variable)	Unadjusted regression coefficient B	Significance p value	95% Confidence interval
a	Age of onset	8.9	− .636	< .001	− .921 to − .351
b	Family	6.7	6.746	< .001	3.227 to 10.265
	History				
	Density				
c	Age of onset	12.5	− .249	< .000	− .82 to − .24
	Family		.197	< .005	1.60 to 8.65
	History				
	Density				

Linear regression analyses used. Dependent variable: Severity of Alcoholism. (a) Predictor: Age of Onset; (b) Predictor: Family History Density ; (c) Predictor: Age of Onset, Family History Density

**Figure 2:**
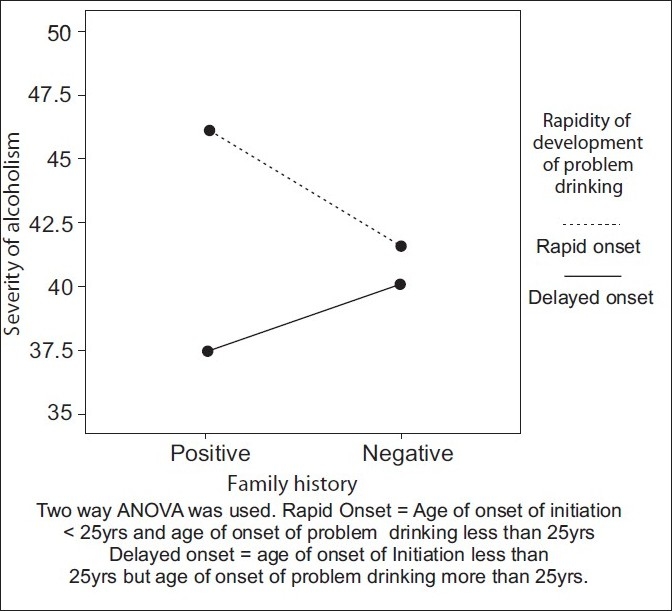
Selecting those with early age of initiation, effect of family history on severity of alcoholism in those with and without early development of problem drinking

We divided the age of onset of initiation into quartiles. The one-way ANOVA test was used to assess the relationship of different quartiles with the mean severity scores. Overall the mean severity scores and age of onset had significant differences between the groups and were statistically significant (F (3,196)=6.15, *P*<.001). A Post hoc Bonferroni test indicated that the mean severity scores significantly differed from the youngest age group (less than and equal to 18 years) as against the oldest age group (>26 years) (*P*<.001) [Table 4]. Two-way ANOVA was used to evaluate the effect of family history on a subgroup of subjects who had age of onset before 25 years and who went into problem drinking before 25years (i.e., showed rapid development of the Problem-drinking). It was seen that age of onset of the Problem-drinking had a significant effect on the severity of alcoholism (F (1,167)=5.75, *P*<.018); whereas, family history status (F (1,167)=.189, *P*>.665) did not have a statistically significant effect. However, there was trend seen between the two factors. [Figure 2]

**Table 4:** Comparing means, standard deviations, and confidence intervals of different quartiles of age of onset with severity of alcoholism

Age of onset	Number	Mean	Std. deviation	95% CI
Less than 18 years	63	45.89	10.044	43.36 to 48.42
19–20 years	43	42.74	12.679	38.84 to 46.65
21–25 years	62	42.16	9.867	39.66 to 44.67
>26 years	32	35.69	12.180	31.30 to 40.08

## DISCUSSION

Few of the previous studies have attempted to use dimensional methods to measure the family history of alcoholism. We have used dimensional and dichotomous measures to acquire the family history of alcoholism. Both measures support a strong relationship of positive family history of alcoholism with severity of alcoholism in the proband. This finding is consistent with a previous study by Stoltenberg *et al*., where seven measures of family history methods were compared and found to be associated with a diagnosis of alcohol dependence.[7] Hill and colleagues[24–26] found that high-risk children with a greater familial density of alcoholism have a higher risk for early alcohol initiation than those with lower familial density of alcoholism, by using a quantitative estimate of familial loading for alcoholism (number of first-degree and second-degree relatives affected). The quantitative dimensional methods help us explore this relationship further. Family history density is an advance in the methodology of family history and is viewed currently as a measure of ‘biopsychosocial’ risk. It takes grandparents and parents into the assessment and excludes aunts and uncles. The author states that “by not including information from aunts and uncles to calculate FHD, even though they are as genetically informative as grandparents, we are underscoring the position that FH measures are not measures of genetic risk, but of biopsychosocial risk. In this context, grandparents are not interchangeable with aunts and uncles because grandparents have greater potential influence. Grandparents establish and maintain the rearing conditions of parents, aunts, and uncles. Therefore, grandparents have a more direct path of influence than do aunts and uncles.” However, this view is yet to be tested in an arguably more familially networked country such as India.

High-risk studies looking specifically at age of onset and severity of alcohol use alone are very few. The concept of the ‘age of onset’ has been a phenomenological advance and the look out for a perfect definition is still a controversy. Most of the authors have a different consensus about the ‘age of onset’ of alcoholism. Latcham determined the age of onset of the Problem-drinking by subtracting the number of years the subject declared he had found his ‘drinking had been a problem’ from his current age.[27] Lee and Diclemente defined age of onset as “the age at which a consistent pattern of heavy alcohol use was established”;[9] Irwin *et al*. defined age of onset as the “age at which subject first met the DSM3R criteria for alcohol abuse or dependence,”[10] and Grant *et al*. defined the age of onset as the “age at which they first started drinking, not counting small tastes or sips of alcohol”.[21] We have used the Grant *et al*. 21 definition for the age of onset of initiation. Despite differing definitions, the trend for higher severity of alcoholism in those with lower age of onset appears to be maintained.

The inverse relationship between the age of onset of initiation with severity of alcoholism is consistent with the previous studies.[28–30] Of the Indian studies, Varma *et al*. does support the idea of a correlation between severity of alcoholism and early age of onset.[11] We have shown that age of onset of initiation is significantly correlated with the severity of alcoholism, even after controlling for the family history of alcoholism, and has a greater effect than family history status alone. This finding is also consistent with previous studies by Grant[21] and Hingson.[31] The study by Hingson *et al*. reports that those who begin drinking before the age of 14 years are more likely to experience alcohol dependence within 10 years of the first drink, even after controlling for sociodemographic data, smoking, illicit drug use, childhood antisocial behavior, depression, and family alcoholism history.[31] The most parsimonious explanation of this would be that the age of onset has a stronger effect on severity than that exerted by the family history. When these two variables are factored in, can this be expected to have implications for Preventive Psychiatry? Can it allow for instituting more intensive education against early exploration of alcohol to high-risk children? So that, by adequately delaying the onset of use, we can explore cutting down the severity of alcoholism in the offspring, if not actually achieve abstinence. Given the public health importance of this possibility, it is worthwhile studying in greater depth, the role of the age of onset in the natural/intervened outcome of the offsprings of subjects with alcoholism. However, the inclusion of a variety of problems, which may go more into the realm of co-morbidity than those that are specifically alcohol-related, obscures the picture.[9] The Twin study by Picken *et al*. has linked these two aspects together and further, with inherited factors, in contrast to Robins *et al*. who caution that early onset of drinking may be an indicator of other psychiatric disorders and may not be a direct factor in the subsequent problem manifestation.[32,33]

We also found that the effect of age of onset of initiation on the severity of alcoholism was significantly associated with the youngest age group (<=18 years) [Table 4]. Beth A. Reboussin in their study reported that young adulthood (ages 18–20 years) was significantly associated with a regular Problem-drinking and suggested that the underage Problem-drinking was most strongly characterized by heavy drinking behaviors that could emerge in late adolescence.[34] They also cautioned that the opportunity for preventing regular drinking might be narrower than what was previously thought, due to the heterogeneous nature of the underage drinking. Tuuli Pitkänen who did a prospective study on the relationship between age of onset of drinking and the use of alcohol in adulthood also found that a low age of onset of drinking was a significant risk factor for high consumption of alcohol and Problem-drinking in adulthood.[30] In their sample, they found that the participants, who initiated drinking prior to the age of 14 years, scored higher in adult alcohol use indicators than individuals who began drinking at the of age 18 years or later (the legal age limit). These studies emphasized that underage drinking be considered seriously and preventive measures begun early. However, studies such as the one by Prescott and Kendler have claimed that delaying the age of onset of drinking would not prevent severe alcoholism. This was based on their analysis of monozygotic and dizygotic twins using bivariate twin models. The authors state that “the results of twin-pair analyses suggest that all of the association between early drinking and later AD is due to familial sources, which probably reflect both shared environmental and genetic factors.” These results suggested that the association between drinking onset and diagnosis was noncausal, and attempts to prevent the development of AD by delaying drinking onset were unlikely to be successful.[35] This argument was more about the independent valence of the phenomenology of age of onset. However, the significance of the relationship in the presence of family history was not disputed by the authors. Given this, one can surmise that a community sample of subjects based on age of onset may not necessarily show alcoholism later, but if they are also selected for a positive family history, they may potentially identify those at significant risk. On the other hand, when we evaluated the effect of family history on the relationship between the severity of alcoholism and age of onset of initiation, in a subgroup of subjects who had an early age of initiation and early onset of Problem-drinking, we found that the family history status did not play a significant role in this relationship, although there was a trend toward the same. These issues need to be studied further in larger community samples.

Current Genetics literature suggests that 40–56 % of the variance in frequency and amount of alcohol used by adults is contributed by genetic factors; also it is suggested that this genetic influence is consistent across the adulthood.[36–38] However, looking at it differently, the amount of variance in the severity of alcoholism explained by the age of onset has ranged from 5 to 9% and family history contributed to 2.9 to 8.3%.[2,6] A variety of shared environmental measures are said to mediate further risk.[32] In our study we found that age of onset contributed 8.9% of the variance in severity of alcoholism, whereas, family history density contributed 6.7%. Despite differences in the Indian culture, legal prohibitions, alcohol availability, and family environment, our study shows that the findings are consistent with western studies done 10 - 15 years ago.[6] Given that shared environmental factors are likely to be higher in the more ‘joint’ Indian families, the findings become very significant. This justifies a further analysis of community factors and family history in the Indian context.

Some of the strengths of the study are highlighted below. This study has been done in a general hospital setting, where a wide spread of severity can be anticipated; previous studies were done in de-addiction units and alcoholic clinics, where the severity scores could be skewed. The methodology of assessment has been clearly explained. We have used both dichotomous and continuous methods of assessments in both the independent variables. One of the major strengths of the study is the use of a reliable method of assessment of Family History, which has been confirmed with four close relatives. According to Gershon and Gurhoff the reliability of interviewing diagnosing relatives, improves from 15 to 64%, when information is collected from four close relatives.[39]

The findings of the study have to be interpreted in the light of the following limitations. First, the screening criteria taken by us using a cut off score of eight in AUDIT may have been too high and missed subjects who could have lower severity scores (48 subjects were excluded from the study on this account). Some of the studies have used the CAGE questionnaire as the screening tool for problem drinking; whether CAGE should be considered because of its low threshold in screening for problem drinkers[30,40] in such research designs, needs consideration. Second, the use of SADQ as a severity scale, like most other severity scales in alcoholism, has not been extensively used or validated in the Indian context. Third, whether alcoholism was primary or secondary to other illnesses in the close relatives of alcoholics, has not been explored.

## CONCLUSION

Using general hospital samples and a more refined methodology, we have demonstrated the effects of family history and age of onset on the severity of alcoholism. The variance explained by these two measures on the severity is not very different from that reported one to two decades ago from western countries with different family structures. Preventive potential by focusing on children of alcoholics has been highlighted. Larger community samples would help confirm our findings. Studies looking at family history and outcomes also have to focus on the relationship between the family history of alcoholism in extended family members and the phenomenology of drinking in index subjects in familially networked societies, such as in India. There is a need to continue studies on children of alcoholics in greater detail, using the currently available behavioral[41] and endogenous markers[8,42,43] in a prospective manner, to elucidate the mechanisms of the risk-outcome relationship. These are the foci of our current efforts.
